# Chemical Structure and Localization of Levan, the Predominant Fructan Type in Underground Systems of *Gomphrena marginata* (Amaranthaceae)

**DOI:** 10.3389/fpls.2018.01745

**Published:** 2018-12-04

**Authors:** Emanuela O. Joaquim, Adriana H. Hayashi, Luce M. B. Torres, Rita C. L. Figueiredo-Ribeiro, Norio Shiomi, Fernanda S. de Sousa, João H. G. Lago, Maria A. M. Carvalho

**Affiliations:** ^1^Programa de Pós-graduação em Biodiversidade Vegetal e Meio Ambiente, São Paulo, Brazil; ^2^Núcleo de Pesquisa em Fisiologia e Bioquímica, Instituto de Botânica, São Paulo, Brazil; ^3^Núcleo de Pesquisa em Anatomia, Instituto de Botânica, São Paulo, Brazil; ^4^Department of Food and Nutrition Sciences, Graduate School of Dairy Science Research, Rakuno Gakuen University, Ebetsu, Japan; ^5^Instituto de Ciências Ambientais, Químicas e Farmacêuticas, Universidade Federal de São Paulo, São Paulo, Brazil; ^6^Centro de Ciências Naturais e Humanas, Universidade Federal do ABC, Santo André, Brazil

**Keywords:** Amaranthaceae, soluble carbohydrates, plant anatomy, *campos rupestres*, fructo-polysaccharides

## Abstract

*Gomphrena marginata* Seub. (Amaranthaceae) is an endemic species from Brazilian *campos rupestres* with a fructan accumulating underground reserve system. Analyses of high performance anion exchange chromatography (HPAEC–PAD) revealed the presence of the soluble carbohydrates glucose, fructose, sucrose, 1-kestose, 6-kestose, nystose and fructans with degree of polymerization (DP) up to approximately 40 fructose units. Data of ^1^H and ^13^C Nuclear Magnetic Resonance (NMR) spectroscopy, including Heteronuclear Single-Quantum Correlation (HSQC) and Heteronuclear Multiple-Bonds Correlation (HMBC) showed the presence of β (2,6) linkages, characteristic of the linear molecule of levan-type fructan(2,6). These results confirmed previous studies suggesting that the reserve carbohydrate in the underground system of this species was levan-type fructans, similar to that of *G. macrocephala*. Structural analyses of the thickened underground system using light microscopy revealed a mixed origin system consisting mainly of a gemmiferous tuberous root with the upper region formed by short branched stems, both presenting vascular cylinders with unusual growth patterns. Fructan spherocrystals were visualized under polarized light and scanning electron microscopy (SEM) mostly in the cortex and vascular cylinder in both thickened stem and root. In addition to data reported in the literature concerning the occurrence of fructans in the Amaranthaceae, the results presented here suggest that fructans are a trait in this family while the levan-type fructan prevail in *Gomphrena* species.

## Introduction

*Gomphrena marginata* Seub. (Amaranthaceae) is an endemic species from Brazilian *campos rupestres*, specifically found in Minas Gerais State (Flora do Brasil 2020, 2018), listed as a rare species in Brazil (Giulietti et al., 2009). *Campos rupestres* occur at high altitudes with rock outcrops, sandy and nutritionally poor soils (Benites et al., 2007), with well distinct dry winters and wet summers and recurrent fire (Silveira et al., 2016). Due to these particular characteristics, a number of species found in *campos rupestres* present adaptations, such as thickened underground organs, allowing survival under unfavorable environmental conditions. Some of these species store considerable amounts of carbohydrates, in some cases constituted of fructans (Moraes et al., 2016; Joaquim et al., 2018).

Fructans are linear or branched polymers of fructose derived from sucrose, containing one internal or terminal glucose (Hendry and Wallace, 1993). They are classified into five structurally different classes depending on the trisaccharide that originates them, the glycosidic linkages between the fructose units and the presence of branching. Inulin, a linear molecule with β (2,1) linkages and based on the trisaccharide 1-kestose, occurs mainly in eudicot species; phlean or levan, a linear molecule with β (2,6) linkages, based on 6-kestose; graminan, a branched molecule with β (2,6) and β (2,1) linkages; the neoseries of inulin with β (2,1) linkages, and the neoseries of levan with β (2,6) linkages, both based on neokestose, with an internal glucose (Carvalho et al., 2007). Fructans also differ from one another by their degree of polymerization (DP), the smallest being the trisaccharides (DP3) and the largest reaching DP 50 in Asteraceae and DP 300 in Poaceae (Yamamoto and Mino, 1985).

Levans are present in a wide variety of organisms: bacteria, fungi, algae, and plants (Donot et al., 2012). In plants, levan is commonly found in grasses such as *Dactilys glomerata* and *Phleum pratense* (Carvalho et al., 2007). A recent study with high performance anion exchange chromatography with pulsed amperometric detection (HPAEC/PAD) suggested that the carbohydrates accumulated in underground systems of *G. marginata* are levan-type fructans (Joaquim et al., 2018). This compound is atypical in eudicotyledonous and reported, until now in *G. macrocephala* (Shiomi et al., 1996) and *Pachysandra terminalis* (Buxaceae), this last one accumulating graminan, besides levan-type fructan (Van den Ende et al., 2011). Among Amaranthaceae, apart from levan in *G. macrocephala*, inulin-type fructan was reported in roots of *Pfaffia glomerata* (Caleffi et al., 2015) and in *Achyrantes bidentata,* the fructan was chemically identified presenting both β (2,6) and β (2,1) linkages (Wang et al., 2015).

Levans have a wide range of potential applications in medical, pharmaceutical, chemical and food industries (Srikanth et al., 2015). Studies have shown that the addition of levan to the diet prevents atherosclerosis in rats, by reducing oxidative stress (Belghith et al., 2012), obesity and hyperlipidemia (Kang et al., 2004). Levans also exhibit anti-inflammatory and anti-cancer activities (Sarilmiser and Oner, 2014) and are recognized for the bifidogenic effect, considered a prebiotic compound on human organism (Adamberg et al., 2015). Numerous species of Amaranthaceae are used in folk medicine (Fank-de-Carvalho et al., 2012) and most of them present underground reserve systems with anomalous secondary growth (Metcalfe and Chalk, 1950) responsible for the thickness of the organ. *Gomphrena* is one of the most widespread genus of Amaranthaceae, with 120 species, of which approximately 46 occur in the Brazilian cerrado and *campos rupestres* (Siqueira, 1991).

Considering the structural diversity of fructans found so far in Amaranthaceae species and the high fructan concentration in the underground system of *G. marginata* (Silva et al., 2013) the aim of this study was to characterize the chemical structure and the tissue distribution of the fructan polymers in this species.

## Materials and Methods

### Plant Material

Six plants of *G. marginata* Seub. were collected at Serra de Itacambira in the Espinhaço Mountain Range (16°59′47^′′^S, 43°20′01^′′^W – Minas Gerais State, Brazil). Three specimens were used for anatomical studies and the remaining three, for carbohydrates analyses. Voucher herbarium specimen was deposited in the SP Herbarium (Instituto de Botânica, Brazil), under number SP 441822.

### Carbohydrate Analyses

For carbohydrates analyses samples of the underground system were frozen in liquid N_2_. Approximately 20 g were used for carbohydrate extraction. The samples were boiled in 80% aqueous ethanol for 3 min for enzyme denaturation and preservation of fructan structure. The tissues were ground and the homogenates were subsequently kept in a water bath at 80°C for 15 min and centrifuged at 1000 *g* for 15 min. This procedure was repeated twice. The residues were subjected to water extraction twice at 60°C for 30 min and filtered under vacuum (Carvalho et al., 1998). For HPAEC/PAD analysis, samples of the ethanol supernatants and water filtrates, constituting the total soluble carbohydrate extract, were pooled and treated as described below, whereas for Nuclear Magnetic Resonance (NMR) analyses, only the filtered water extract, constituting the polysaccharide fraction was used and treated as described below.

### High Performance Anion Exchange Chromatography

Ethanol supernatants and water filtrates were pooled, concentrated and de-ionized through ion exchange columns (Dowex 1 × 8 – 200 – chloride form and 50 × 8 – 100 – hydrogen form) (Carvalho et al., 1997). Identification of soluble carbohydrates was performed by high performance anion exchange chromatography with pulsed amperometric detection (HPAEC–PAD) and a 2 mm × 250 mm CarboPac PA-1 column on an ICS 3000 Dionex System (Asega et al., 2008), using the software Chromeleon 7.2.6. The gradient was established by mixing eluant A (150 mmol L^-1^ NaOH) with eluant B (500 mmol L^-1^ sodium acetate in 150 mmol L^-1^ NaOH) as follows: 0–2 min, 25 mmol L^-1^; 2.1–8.5 min, 50 mmol L^-1^; 8.6–10 min, 75 mmol L^-1^; 10.1–28 min, 100 mmol L^-1^; 28.1–30 min, 500 mmol L^-1^; and 30.1–40 min, 25 mmol L^-1^, using a flow rate of 1 ml min^-1^ through the column. The applied PAD potentials for E1 (0–0.4 s), E2 (0.41–0.42 s), E3 (0.43 s), and E4 (0.44–1.00 s) were 0.1, -2.0, 0.6, and -0.1, respectively.

### NMR Analyses

The water polysaccharide fraction was submitted to a clean-up by ion exchange columns, as described for HPAEC/PAD above, followed by lyophilization. The purity of the polymeric sample was determined by HPAEC/PAD and ^13^C and ^1^H NMR. Subsequently, the sample was submitted to Heteronuclear Single-Quantum Correlation (HSQC) and Heteronuclear Multiple-Bonds Correlation (HMBC) analyses in spectrometer Varian INOVA, operating at 500 and 125 MHz to ^1^H and ^13^C nucleus, respectively, using MestReNova Version: 6.0.2.5475 2009 Mestrelab Research S.L. All spectra were recorded using D_2_O as solvent. Results of the chemical shifts were shown as delta (δ) in parts per million (ppm), and the simple (s), double (d), triple (t), multiple (m) signals, and measurements of coupling constants (*J*), in Hz. For the two-dimensional HMBC, results are expressed as ^2^*J*_H/C_ or ^3^*J*_H/C_, meaning that ^1^H and ^13^C cross peak at 2 or 3 chemical bonds, and for HSQC the results are expressed as *J*_H/C,_ a direct correlation between ^13^C and ^1^H.

### Anatomical Analyses

For light microscopy, the underground systems of *G. marginata* were fixed in FAA 70 (formaldehyde, acetic acid, and 70% ethanol in a ratio of 1:1:18 by volume) (Johansen, 1940). For visualization of fructan spherocrystals under polarized light, samples were maintained in 70% ethanol before free-hand cross sectioning, while confirmation of fructan presence was done by thymol-sulphuric acid reagent (Johansen, 1940). Lugol reagent test was used to access the presence of starch crystals (Berlyn and Miksche, 1976).

For infiltration in hydroxy-ethyl-methacrylate resin (Leica Historesin), the samples were dehydrated in a graded ethylic series. Sections (7 mm thick) were cut on a rotary microtome and stained with 0.05% toluidine blue O (Sakai, 1973) in phosphate-citrate buffer at pH 4.5. Images were captured digitally with an Olympus BX53 microscope coupled with an Olympus Q-Color 5 camera, using the software Image-Pro Express 6.3 (Media Cybernetics).

For scanning electron microscopy (SEM), samples were fixed in Karnovsky solution (Karnovsky, 1965) and dehydrated in a graded acetone series, dried in a CO_2_ critical point dryer, mounted on aluminum supports and coated with gold. Observations were performed using a Philips XL Series XL 20 microscope.

## Results

### Carbohydrates Analyses

**FIGURE 1:**
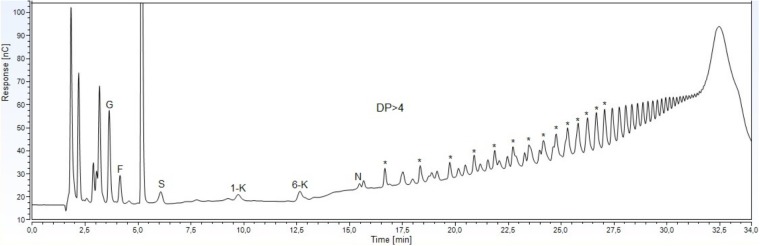
HPAEC-PAD profile of soluble carbohydrates from underground storage system of *Gomphrena marginata*. Glucose (G), fructose (F), sucrose (S),1-kestose (1-K), 6-kestose (6-K), nystose (N), and fructans with degree of polymerization higher than 4 (DP > 4). The asterisks indicate components of the levan series.

HPAEC–PAD analysis of the carbohydrates extracted from the underground system indicated the presence of glucose, fructose, sucrose, the DP3 1-kestose and 6-kestose, of the inulin and levan series, respectively, and the DP4 1,1, kestotetraose (nystose), based on comparison with known external standards (Figure 1). The identities of the components of the prevalent homologous series were deduced using levans of *Dactylis glomerata* as standard, since the main peaks of the polysaccharides coincided with the peaks of this standard series.

**FIGURE 2:**
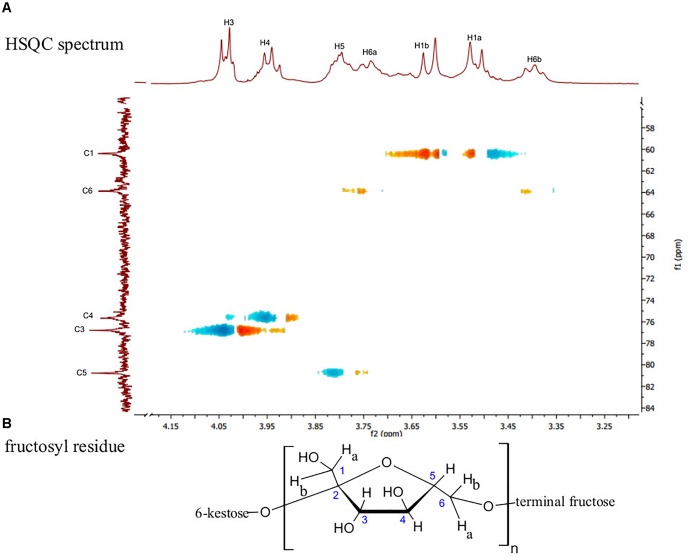
**(A)** HSQC spectrum in D_2_O showing the direct connectivity between the ^13^C and ^1^H (*J*_H/C_) of the fructosyl residue of levan-type fructan polysaccharides from *G. marginata* stored in the underground system, and **(B)** Fructosyl residue.

**FIGURE 3:**
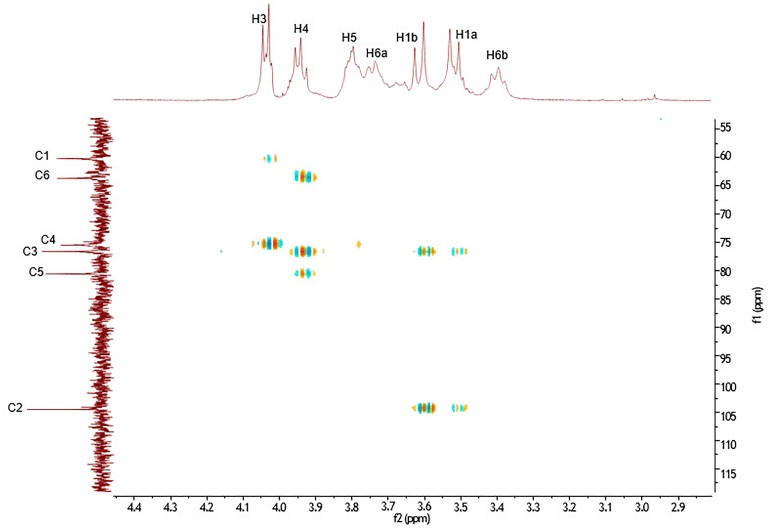
HMBC spectrum in D_2_O showing the correlations (^2^*J*_H/C_ and ^3^*J*_H/C_) between the ^1^H and ^13^C of the fructosyl residue of levan-type fructan polysaccharides from *G. marginata* stored in the underground system.

The ^13^C NMR spectral data of *G. marginata* polymer (Table 1) showed six signals at δ_C_ 104.69, 80.76, 76.79, 75.67, 63.88, and 60.39, referring to carbons of the fructosyl residue. Among the peaks, signal at δ_C_ 104.69 was attributed to the β configuration at anomeric carbon (C-2) while those at δ_C_ 60.39 and 63.88 were assigned to methylene group (C1 and C6). Also, ^13^C NMR data showed a signal at δ_C_ 80.76 (C5) which was assigned to furanose, and signals at δ_C_ 75.67 and 76.79 corresponding to oxymethinic groups of the fructosyl residue (C-4 and C-3) (Table 1). The ^1^H NMR spectrum showed peaks at δ_H_ 3.39 (t, *J* = 8 Hz, H-6a), 3.51 (d, *J* = 12.1 Hz, H-1a), 3.61 (d, *J* = 12.1 Hz, H-1b), 3.73 (m, H-6b), 3.76 (m, H-5), 3.94 (t, *J* = 7.5 Hz, H-4) and 4.03 (d, *J* = 8.4 Hz, H-3). All of these data were obtained by the analysis of HSQC (Figure 2A) and HMBC (Figure 3). The HSQC spectrum, used to assign the hydrogens directly linked to their respective carbons (J_H/C_), showed cross peaks between H6a and H6b/C6, H1a and H1b/C1, H5/C5, H4/C4, H3/C3 and no cross peaks for C-2, confirming its quaternary anomeric carbon character (Table 2). HMBC analysis showed ^1^H and ^13^C atoms correlated within 2 (^2^*J*_H/C_) or 3 (^3^*J*_H/C_) chemical bonds. Thus, HMBC spectrum showed cross peaks between H1a and H1b/C3, H3/C1, H3/C4, H4/C3, H4/C5, H4/C6, H5/C4 and H1a and H1b/C2 (Table 3). Data of ^1^H and ^13^C NMR, HSQC and HMBC are compatible with data of β (2,6) linkages, characteristic of the fructosyl residue of levan-type fructans (Figure 2B), in accordance with the literature.

**Table 1:** ^13^C NMR chemical shifts (ppm) data of the fructosyl residue of levan-type fructan (β 2,6) from *Gomphrena marginata* underground storage systems and of levans and inulin described in the literature.

Chemical shifts ^13^C NMR (ppm)					
**Carbon atom**	***Gomphrena marginata***	***Gomphrena macrocephala* (Shiomi et al., 1996)**	***Bacillus licheniformis* (Dahech et al., 2013)**	***Chromohalobacter salexigens* (Husseiny et al., 2015)**	***Pfaffia glomerata* (Caleffi et al., 2015)**

	**Levan**	**Levan**	**Levan**	**Levan**	**Inulin**
C-1	60.3	60.6	59.7	60.7	63.7
C-2	104.6	104.9	104.1	105	106.1
C-3	76.7	76.9	76.1	77.1	79.8
C-4	75.7	75.9	75.1	76	77.2
C-5	80.7	81.0	80.2	81.1	83.9
C-6	63.8	64.1	63.3	64.1	65.0

**Table 2:** HSQC chemical shifts data (δ or ppm) in D_2_O of the fructosyl residue of levan-type fructan polysaccharides from *G. marginata* underground storage system.

Chemical shifts								
**Atoms**	**H1a/C1**	**H1b/C1**	**C-2**	**H3/C3**	**H4/C4**	**H5/C5**	**H6a/C6**	**H6b/C6**
^1^H	3.51	3.61	–	4.03	3.94	3.76	3.73	3.39
^13^C	60.3	60.3	104.6	76.7	75.7	80.7	63.8	63.8

**Table 3:** HMBC chemical shifts (δ in ppm) in D_2_O data of the fructosyl residue of levan-type fructan polysaccharides from *G. marginata* underground storage system.

Chemical shifts (ppm) ^2^*J*_H/C_ and ^3^*J*_H/C_						
**Atoms**	**C1 (^3^*J*_H/C_)**	**C2 (^2^*J*_H/C_)**	**C3 (^3^*J*_H/C_)**	**C4 (^2^*J*_H/C_)**	**C5 (^2^*J*_H/C_)**	**C6 (^3^*J*_H/C_)**
H1a		3.61/104.6	3.61/76.7			
H1b		3.51/104.6	3.51/76.7			
H3	4.03/60.3			4.03/75.7		
H4			3.94/76.7		3.94/80.7	3.94/63.8
H5				3.76/75.7		

### Anatomy of the Underground System

**FIGURE 4:**
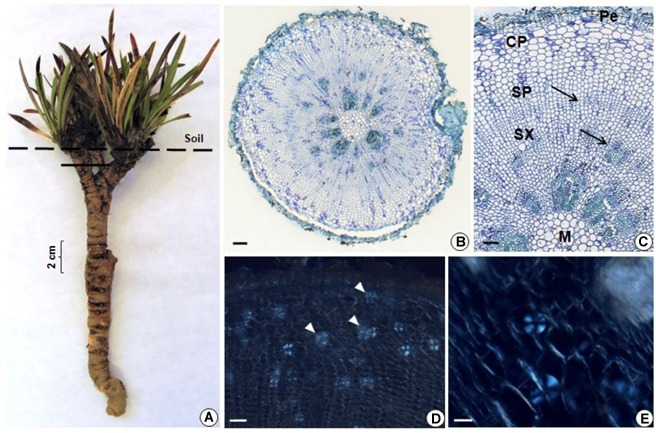
**(A)** General view of *G. marginata*; continuous line indicates the sectioned region. **(B–E)** Transverse sections of the stem. General **(B)** and detailed **(C)** view, showing periderm (Pe), cortical parenchyma (CP), secondary phloem (SP), secondary xylem (SX), cambia (arrows) and medulla (M). Fructan spherocrystals (arrowheads) visualized under polarized light in the parenchyma cells of the cortex, in the secondary phloem and xylem **(D)** and medulla **(E)**. Scale bars: 200 μm **(B)**, 100 μm **(C,D)**, and 50 μm **(E)**.

**FIGURE 5:**
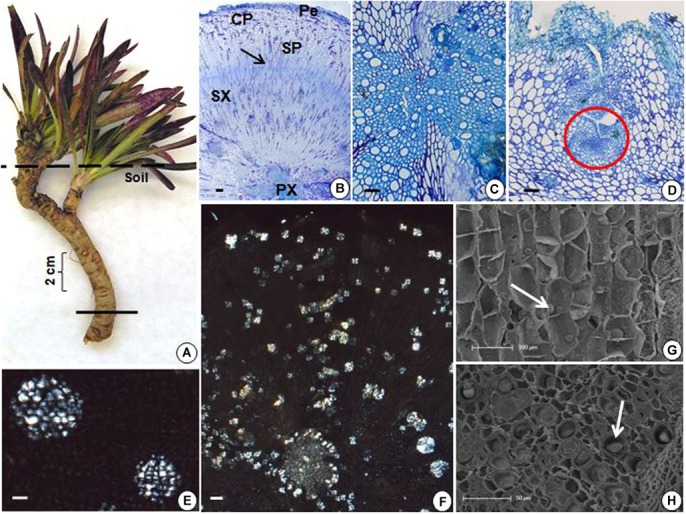
**(A)** General view of *G. marginata*; continuous line indicates the sectioned region. **(B–H)** Transverse sections of tuberous root. **(B)** General view showing periderm (Pe), cortical parenchyma (CP), secondary phloem (SP), secondary xylem (SX), cambium (arrow), and primary xylem (PX). **(C)** Detail of the central region of the tuberous root. **(D)** Presence of bud (circle). **(E,F)** Fructan spherocrystals visualized under polarized light, forming at times clusters **(E)** in the parenchyma cells of the cortex, in the secondary phloem and xylem **(F)**. **(G,H)** Scanning electron micrographs with fructan spherocrystals (arrows) in the parenchymatic cortex **(G)** and inside the vessel elements **(H)**. Scale bars: 200 μm **(B,F)**, 100 μm **(D,G)**, and 50 μm **(C,E,H)**.

The underground reserve system of *G. marginata* has a mixed origin consisting predominantly of a vertical fleshy tuberous root that grows downward into the soil (Figures 4A, 5A). However, the upper region of the underground system consists of some short branched stems, with leaves appearing only above the soil surface (Figure 4A). The stems and tuberous roots of the analyzed specimens presented secondary structure (Figures 4B,C, 5B). Periderm covers the entire underground system and the cortex presents few layers of parenchymatic cells (Figures 4B,C, 5B). The vascular cylinder shows an unusual pattern, with irregular supernumerary cambia, which produces a larger proportion of reserve parenchyma cells outward and small amounts of vascular tissue (Figures 4B,C, 5B). Primary xylem exhibits centrifugal maturation and a medulla in its core, confirming the stem structure in the upper region of the organ (Figure 4C). In the stem, fructan spherocrystals were visualized in the cortex, parenchymatic cells of secondary xylem and phloem, and medulla (Figures 4D,E). Mostly, the underground system presents root structure, confirmed by centripetal maturation of the primary xylem with the presence of metaxylem in the center (Figure 5C). In the tuberous root, buds were observed (Figure 5D) and fructan spherocrystals were visualized in parenchyma cells of the cortex and vascular cylinder (Figures 5F,G), forming clusters at times (Figure 5E). The fructan nature of these crystals was confirmed by the reaction on the crystals, which exhibited the carmine color following treatment with thymol-sulphuric acid (Supplementary Figure 1). Spherocrystals were also visualized inside the vessel elements (Figure 5H). Lugol tests were negative for starch in both stem and root.

## Discussion

Previous chromatographic studies with *G. marginata* using HPAEC/PAD suggested that levan-type fructans are the carbohydrates accumulated in the underground system of this Amaranthaceae (Joaquim et al., 2018). Nevertheless, in this study, the detection of 1-kestose and nystose, and probably of the intermediate small peaks between the main levan peaks indicate a minor presence of inulin oligosaccharides. ^13^C NMR chemical shifts of the fructosyl residue of the polysaccharide from reserve tissues of *G. marginata* showed similarity with characteristic chemical shifts of levan-type fructans reported for *G. macrocephala* (Shiomi et al., 1996) and for some species of microorganisms (Table 1). Levans are well spread among plant species, especially in Poaceae. This fructan type was identified by ^13^C NMR analyses in *Lolium perenne* (Tomasic et al., 1978), *Festuca arundinaceae* (Carpita et al., 1991), *Poa trivialis* and *Phalaris arundinacea* (Hammer and Morgenlie, 1990). However, no data of ^13^C and ^1^H HSQC or HMBC spectral analyses for levan-type fructans of other Angiosperms are reported. Recent spectral data (uni and two-dimensional NMR) in the literature refer to levans from microorganisms, such as *Pseudomonas fluorescens* (Jathore et al., 2012), *Lactobacillus reuteri* (Sims et al., 2011), *Bacillus subtiles* (Shih et al., 2005), *Chromohalobacter salexigens* (Husseiny et al., 2015), and *Bacillus licheniformis* (Dahech et al., 2013), comparable to those obtained for *G. marginata*.

The present study was based on the technologies of HPAEC/PAD and NMR to characterize the fructan type and the linkage type between the fructosyl units, respectively. Stahl et al. (1997) used HPAEC/PAD and Matrix-Assisted Laser Desorption/Ionization Mass Spectrometry (MALDI-MS) to do a comparative study of commercial inulin and fructans of onion bulbs, and showed that although both are complementary techniques, they present limitations since the characterization of the linkage type cannot be resolved.

In roots of *P. glomerata* (Amaranthaceae), the unequivocal presence of inulin-type fructan was confirmed by NMR analyses (Caleffi et al., 2015). In *A. bidentata* and *Cyathula officinalis*, well known medicinal species of Amaranthaceae from China, the identified fructans present both β (2,6) and β (2,1) linkages (Ying-Hua et al., 2004; Wang et al., 2015). Furthermore, chromatographic analyses indicated the accumulation of levan-type fructans in underground systems of *G. agrestis* (Joaquim et al., 2018), *G. monquinii*, *G. scapigera*, and *G. decipiens* (Silva, 2001). The information above, in addition to the unequivocal presence of levans in *G. macrocephala* (Shiomi et al., 1996) and *G. marginata*, suggests that fructan accumulation in Amaranthaceae could be a family trait, while the type of fructan stored may differ depending on the genera.

Fructans have been extensively studied in monocotyledonous and among these, a structural diversity is found (Pollock and Cairns, 1991). In Poaceae, for example, graminan-type fructans occur in cereals such as *Triticum aestivum* and *Hordeum vulgare* (Bancal et al., 1992), while levan-type is found in *D. glomerata* (Chatterton et al., 1993) and *Phleum pratens*e (Tamura et al., 2009) and the neoseries in *L. perenne* (Pavis et al., 2001). Among the Amaryllidaceae, *Allium cepa* accumulates the neoseries (Yamamori et al., 2015) and among the Agavaceae, a complex mix of graminans and branched neo-fructans, named agavins (Mancilla-Margalli and López, 2006; Arrizon et al., 2010) are found. In eudicotyledonous, inulin is common in the Asteraceae (Moraes et al., 2016 and refs therein, Joaquim et al., 2018); however, the presence of other types of fructans in Amaranthaceae (Shiomi et al., 1996; Ying-Hua et al., 2004; Wang et al., 2015) and Buxaceae (Van den Ende et al., 2011, 2016), indicate that a variety of fructan structures occurs in eudicots as well. Van den Ende (2013) suggested that the capacity to synthesize fructans emerged at least 4 times in the evolutionary process: in Asterales, which store inulin-type fructans; in Poales, graminan, levan and the neoseries; Asparagales and Agavoideae, neoseries type; and in the basal eudicot *Pachysandra terminals* (Buxaceae) which accumulates graminan and levan. From an evolutionary point of view, fructans in Amaranthaceae are still far from being widely investigated, however, the studies until now point to a diversity of fructan types within this family.

Concerning the identification and visualization of fructan spherocrystals, the method used in this study was described specifically for inulin (Johansen, 1940); however, in underground systems of *G. marginata*, where levan-type fructans are predominantly accumulated, as determined by NMR analyzes, spherocrystals were also clearly visualized under polarized light. In order to certify that the visualized birefringent spherocrystal structures formed after treatment with ethanol were fructans, the lugol reagent (Berlyn and Miksche, 1976) was firstly used to exclude the possibility of starch presence, since both polysaccharides form typical “Malta cross” structures when visualized under polarized light. Secondly, we performed a test to detect the presence of fructose polymers (fructans), the thymol-sulphuric acid reagent (Johansen, 1940). The first one was negative for starch, and the second confirmed the presence of fructan.

Fructan spherocrystals were also visualized in tuberous root tissues of the levan accumulating species *G. macrocephala* (Vieira and Figueiredo-Ribeiro, 1993). In *G. marginata,* spherocrystals were observed mainly in the vascular cylinder, and inside the vessel elements. Similar localization was reported in *G. macrocephala* (Vieira and Figueiredo-Ribeiro, 1993) and in different types of underground systems of various Asteraceae species (Tertuliano and Figueiredo-Ribeiro, 1993; Oliveira et al., 2013; Joaquim et al., 2014). In the Asteraceae *Aldama tenuifolia*, inulin spherocrystals were visualized in the lumen of the internal secretory spaces of thickened roots in addition to vascular tissues (Silva et al., 2014).

The underground reserve system of *G. marginata* is mainly a tuberous root, capable of resprouting due to the presence of buds, also described in *Pfaffia gnaphalioides* (Grosso, 2007) and in tuberous roots of *Vernonia brevifolia* (currently *Lessingianthus brevifolius*) (Hayashi and Appezzato-da-Glória, 2007) and *V. oxylepis* (Vilhalva and Appezzato-da-Glória, 2006), both inulin accumulating Asteraceae species. The presence of buds in belowground organs allows the regeneration of the aerial parts after drought, fire or any other environmental disturbance (Klimesová and Klimes, 2007), while the reserve compounds therein supplies energy and carbon to guarantee resprouting, growth, and ultimately, species survival.

The secondary structures of roots and stems observed in *G. marginata* show an unusual or anomalous thickening of the vascular cylinder due to the high activity of supernumerary cambia, resulting specially in the formation of abundant reserve tissues. This anomalous thickening is a common phenomenon in the Amaranthaceae (Metcalfe and Chalk, 1950; Menezes et al., 1969; Grosso, 2007; El-Ghamery et al., 2015; Sá et al., 2016) and Chenopodiaceae (Metcalfe and Chalk, 1950; Krumbiegel, 1998). In *G. albiflora*, root secondary growth was also considered atypical (Jáuregui et al., 2014) as the authors showed that the successive rings are formed from a lateral meristem producing parenchyma outward, supernumerary cambia and parenchyma inward.

Due to a number of shared characteristics with respect to morphology, anatomy, phytochemistry and phylogeny, Amaranthaceae *sensu lato* included the Chenopodiaceae (Kadereit et al., 2003). Curiously, a fructan exohydrolase enzyme (6-FEH) was purified, cloned and characterized from the tuberous roots of the Chenopodiaceae sugar beet (*Beta vulgaris*), a sucrose storing plant (Van den Ende et al., 2003). This unexpected presence in a non-fructan-storing plant, in addition to the above mentioned traits, could be an evidence of the evolutionary relationship between these two families.

## Conclusion

In conclusion, *G. marginata* shows an unusual secondary growth which produces abundant parenchyma cells, in which fructans can be stored to provide energy for regeneration of aerial parts, after drought, fire, abiotic stresses and other environmental disturbances frequent in *campos rupestres*. Data concerning the variety of fructan structures occurring in the Amaranthaceae highlight the fact that eudicots, and not only monocots, present a diversity of fructan types. In this study, the predominance of levan-type fructans in *G. marginata* underground system (stem and root) was confirmed, demonstrating not only that fructan metabolism is a characteristic of the Amaranthaceae, but also that a structural diversity of fructans occurs in members of this family.

## Author Contributions

EJ, AH, RF-R, and MC conceived and designed the research. EJ carried out all the biochemical and anatomical analyses. AH and EJ interpreted the anatomical results. LT, NS, JL, and FS were responsible for NMR analysis. LT, NS, JL, and EJ contributed to interpretation of chemical data. MC, EJ, AH, LT, and RF-R analyzed the data altogether and wrote the manuscript. All authors read, revised, and approved the final text.

## Conflict of Interest Statement

The authors declare that the research was conducted in the absence of any commercial or financial relationships that could be construed as a potential conflict of interest.
