# Supplementary material for: Chemical Structure and Localization of Levan, the Predominant Fructan Type in Underground Systems of Gomphrena marginata (Amaranthaceae)
Source: Front Plant Sci. 2018 Dec 4;9:1745. doi: 10.3389/fpls.2018.01745 (PMC6288709; doi:10.3389/fpls.2018.01745)
Supplement: Supplementary file 1 [file Image_1.pdf]

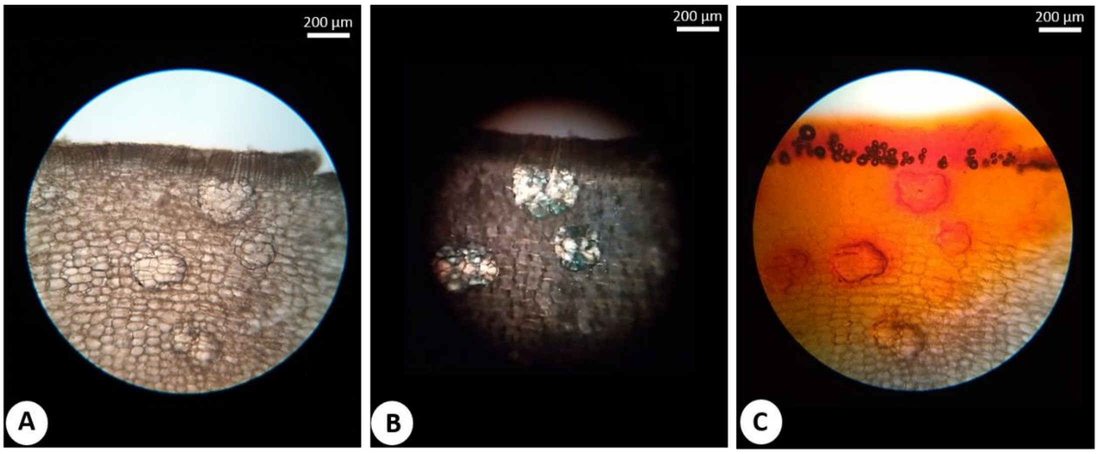

Figure 1. Transverse sections of a *Gomphrena marginata* root, showing clusters of fructan crystals in parenchyma cells of the cortex and vascular cylinder. (A-B) Fructan clusters visualized before treatment with thymol-sulphuric acid reagent under brightfield and polarized light, respectively. (C) Fructan clusters reacting to treatment with thymol-sulphuric acid reagent exhibiting the carmine-red color.
